# Mycobacteria-Specific T Cells Are Generated in the Lung During Mucosal BCG Immunization or Infection With *Mycobacterium tuberculosis*

**DOI:** 10.3389/fimmu.2020.566319

**Published:** 2020-10-22

**Authors:** Juan I. Basile, Ruining Liu, Wenjun Mou, Yu Gao, Berit Carow, Martin E. Rottenberg

**Affiliations:** Department of Microbiology, Tumor and Cell Biology and Center for Tuberculosis Research, Karolinska Institutet, Stockholm, Sweden

**Keywords:** *Mycobacterium tuberculosis*, T cell, lung, resident memory T cells, BCG- immunization

## Abstract

Specific T cell responses are central for protection against infection with *M. tuberculosis*. Here we show that mycobacteria-specific CD4 and CD8 T cells accumulated in the lung but not in the mediastinal lymph node (MLN) at different time points after *M. tuberculosis* infection or BCG immunization. Proliferating specific T cells were found in the lung after infection and immunization. Pulmonary, but not MLN-derived CD4 and CD8 T cells, from *M. tuberculosis*-infected mice secreted IFN-γ after stimulation with different mycobacterial peptides. Mycobacteria-specific resident memory CD4 and CD8 T cells (TRM) expressing PD-1 accumulated in the lung after aerosol infection and intratracheal (i.t.) -but not subcutaneous (s.c.)- BCG immunization. Chemical inhibition of recirculation indicated that TRM were generated in the lung after BCG i.t. immunization. In summary, mycobacteria specific-TRM accumulate in the lung during i.t. but not s.c. immunization or *M. tuberculosis* infection. Collectively our data suggests that priming, accumulation and/or expansion of specific T cells during BCG immunization and *M. tuberculosis* infection occurs in the lung.

## Introduction

Tuberculosis (TB) is the leading cause of infectious death; in 2018, 10 million people developed and 1.5 million patients died from TB (1). The risk of developing TB increases during HIV and *Mycobacterium tuberculosis* co-infection, suggesting that impairment of T cell-mediated immune responses reactivates the asymptomatic infection.

The only TB vaccine, BCG (Bacille Calmette–Guérin) in use since 1921, offers substantial protection of infants against meningeal or miliary TB. However, protection against pulmonary TB in adults is not sufficient. The BCG vaccine, to a large extent and with some exceptions, mitigates only the most severe aspects of infection and exhibits a highly variable efficacy, especially in high-burden areas (2). Developing new and efficacious TB vaccines, a very effective intervention for containing the TB spread, is a critical unmet public health need (3).

The lung is the portal of entry for *M. tuberculosis* and experimental evidence indicates that local T-cell mediated immune defense mechanisms are crucial for successful bacterial control during latent infection with *M. tuberculosis*. Delivering a TB vaccine by aerosol to the respiratory mucosa, as a mimic of natural infection has been shown to augment protection of mice, guinea pigs and macaques as compared to immunization at a distal site (4–9). Following the classical paradigm naïve T cells will be activated in the draining mediastinal lymph nodes (MLNs) after *M. tuberculosis* infection or mucosal immunization. Activated T cells will then egress and migrate to the lung parenchyma for interaction with *M. tuberculosis*-infected phagocytes or differentiate into memory T cells that on re-exposure will mount a rapid and robust response to the pathogen (10). A subset of these, the resident memory cells T cell (T_RM_) reside in the mucosal tissues including the lung and do not recirculate through the blood or the lymphatics (11). Positioned at the site of pathogen invasion, both CD4 and CD8 T_RM_ have been shown to contribute to the early clearance of pathogens such as influenza, LCMV, Sendai, RSV or herpex simplex viruses, or *Listeria sp, Yersinia sp*, and *Chlamydia sp* among others (12–17). T_RM_ are also generated after vaccination or infection with *M. tuberculosis* and have been shown play a role in protection (18). BCG-vaccinated mice sustained protection against *M. tuberculosis* infection even when egress of cells from the secondary lymphoid organs was blocked, suggesting that memory lymphocytes retained in the lung following vaccination were sufficient for protection (19, 20).

Here, we compared the generation of T cells in the lung and MLNs after infection and mucosal and distal BCG immunization. Contrary to expectations, mucosal BCG-vaccination and *M. tuberculosis* infection generated high levels of mycobacteria-specific T cells in the lung, specific T cells remained undetected or very low in the draining mediastinal lymph nodes at all time points tested. Mycobacteria-specific CD4 and CD8 T_RM_ were generated after aerosol *M. tuberculosis* infection and intratracheal (i.t.), but not subcutaneous (s.c.), BCG immunization. Moreover, T_RM_ accumulated in the lung in absence of lymphoid circulation after i.t. BCG immunization, altogether suggesting that upon mucosal immunization or infection mycobacteria specific T cells are generated in the lung. Our data strongly supports mucosal delivery for induction of protective adaptive immune responses against *M. tuberculosis*.

## Materials and Methods

### Ethics Statement

The animals were housed and handled at the Department of Microbiology, Tumor and Cell Biology and the Astrid Fagreus Laboratory, Karolinska Institutet, Stockholm, according to directives and guidelines of the Swedish Board of Agriculture, the Swedish Animal Protection Agency, and the Karolinska Institute (djurskyddslagen 1988:534; djurskyddsförordningen 1988:539; djurskyddsmyndigheten DFS 2004:4). The study was performed under approval of the Stockholm North Ethical Committee on Animal Experiments permit number N397/13 and N487/11. Animals were housed under specific pathogen-free conditions.

### Mice, Infection, and Infectivity Assay

BCG Montreal and *M. tuberculosis* Harlingen were grown in Middlebrook 7H9 (Difco, Detroit, MI) supplemented with albumin, dextrose and catalase.

Eight-10 week-old C57BL/6J mice were used for immunizations or infections. Mice were infected with 250 *M. tuberculosis* Harlingen strain by aerosol during 20 min using a nose-only exposure unit (In-tox Products, Uppsala, Sweden) (21), or immunized s.c. or i.t. with 10^7^ BCG.

The intratracheal aerosol administration was performed using a MicroSprayer® Aerosolizer—Model IA-1C and a FMJ-250 High Pressure Syringe (Penn-Century, Wyndmoor, PA), a device that generates a air-free plume of liquid aerosol directly into the lungs. The tip of the device was inserted near to the carina of the anesthetized animal and 50 μl of BCG suspension containing 10^7^ CFUs was aerosolized into the lungs.

To determine viable numbers of CFUs at time-points post-infection, the right lung of each mouse was homogenized in PBS with 0.05% Tween 80. Ten-fold serial dilutions were made in 0.05% Tween 80 and plated onto Middlebrook 7H11 agar containing 10% enrichment of oleic acid, albumin, dextrose, catalase, 5 μg of amphotericin B per ml and 8 μg/ml polymyxin B grown for 3 weeks at 37°C. Colonies were counted 3 weeks after incubation at 37°C and CFUs determined.

### Flow Cytometry and Intracellular Cytokine Staining

Lungs were removed, mechanically minced into small pieces and digested with 3 mg/ml Collagenase D and 30 μg/ml DNase I for 1 h at 37°C, and single-cell suspensions prepared by filtering lung tissue through 70-μm nylon cell strainers. To further remove impurities cells were loaded in 40/70% Percoll gradient in PBS and centrifuged for 30 min at room temperature. The cells at the interphase were collected and washed. Single spleen cell suspensions were obtained by mechanical disruption, lysis of erythrocytes and straining over a 70-μm nylon mesh. Single cell suspensions were obtained after mechanical disruption of the mediastinal lymph node followed by filtering over a 70-μm nylon mesh.

Lung, lymph node cells and spleen cells were stained for CD3, CD4, CD8, CD69, CD44, CD11a, CD103, PD-1, and KRLG-1 (all from eBioscience) for 30 min 4°C and fixed before acquisition.

To discriminate between tissue-localized and blood-borne cells in an intravascular staining was performed as previously described (22). In short, mice were inoculated i.v. with 3 μg of FITC-labeled anti-CD45.2 (clone 104 BD), sacrificed 3–5 min after i.v. innoculation, and lungs and MLN leukocytes isolated immediately as described. Peripheral blood was sampled for every mouse as a control.

Data were acquired on a LSRII Flow cytometry and analyzed with FlowJo software (Tree star Inc., Ashland, OR).

### Tetramer Staining

MHCII tetramers containing amino acids 1–20 of *M. tuberculosis* ESAT-6 or 240-254 of Ag85B and the MHCI tetramer containing amino acids 4–11 TB10.4 (all from the NIH Tetramer Core Facility) were used for detection of *M. tuberculosis*-specific murine CD4 or CD8 T cells. Single-cell lung or MLN suspensions were stained at saturating concentrations with the tetramers and incubated at 37°C for 1 h for the MHCII tetramers and at 4°C for 30 min for the MHCI tetramer.

### Intracellular Cytokine Staining

For determination of IFN-γ-producing cells, lung and MLN cells from *M. tuberculosis* infected mice were incubated with either 5 μg/ml ESAT6_1−15_, 5 μg/ml TB10.4_4−11_, 20 μg/ml purified protein derivative (PPD) (Statens Serum Institut, Denmark), or 50 ng/ml phorbol myristate acetate (PMA) and 2 μg/ml ionomycin (Sigma) for 6 at 37°C. Brefeldin (10 μg/ml) was added to the cultures the last 4 h of stimulation. Cells were then stained with cell population-specific antibodies, and live/dead staining, fixed, permeabilized using leukocyte permeabilization reagent IntraPrep™ (Immunotech, Marseille, France) and further stained with anti-IFN-γ (eBioscience). Data were acquired on a LSRII Flow cytometry and analyzed with FlowJo software (Tree star Inc., Ashland, OR).

### Antigen-Specific T Cell Labeling *in situ*

The detection of antigen-specific T cells *in situ* was performed modified to a previously described protocol (23). Briefly, sections (ca 200–400 μm) from lungs from *M. tuberculosis*-infected mice kept in PBS containing heparin (100 μg/ml) were obtained using a scalpel. Sections were blocked with 2% normal goat serum, 0.025% triton for 1 h, as all incubations steps at 4°C. After PBS washing, APC-labeled TB10.4 tetramers at a concentration of 0.5 μg/ml with 1% goat serum and 0.5 μg/ml rat anti-CD8a were added to slices and incubated for 4 h. Sections were washed in PBS and then incubated with biotinylated anti-APC Abs and secondary FITC anti-rat antibody both diluted 1:100 overnight. Sections were washed with PBS and then incubated with APC-conjugated streptavidin diluted 1:100 in PBS for 3 h together with DAPI. Slices were washed with PBS and then fixed with PBS-buffered 2% formaldehyde for 2 h at 4°C. Finally, sections were washed three times with PBS and then mounted to slides using Fluoromount™ Aqueous Mounting Medium. Stained sections were analyzed using a confocal microscope (Zeiss LSM 710).

### Statistics

Statistical analysis and graphical representation of data were done using GraphPad Prism 8 software (Grah Pad Prism, San Diego, CA). We have used the Welch's *t*-test, which assumes normal distribution but can be used when the two samples have unequal variances for comparisons (*t-*test). Statistical significance between 3 and more groups was determined using one- or two-way ANOVA. The Welch's test was also used for ANOVA comparisons. We have used used the Turkey's multiple comparisons test or *t-test* with Holm-Sidak correction for multiple comparisons of the same parameters (for example for analyzing kinetics).

## Results

### Mycobacteria-Specific T Cells Accumulate in the Lung but Not the Mediastinal Lymph Node During BCG Immunization or Infection With *M. tuberculosis*

**Figure 1:**
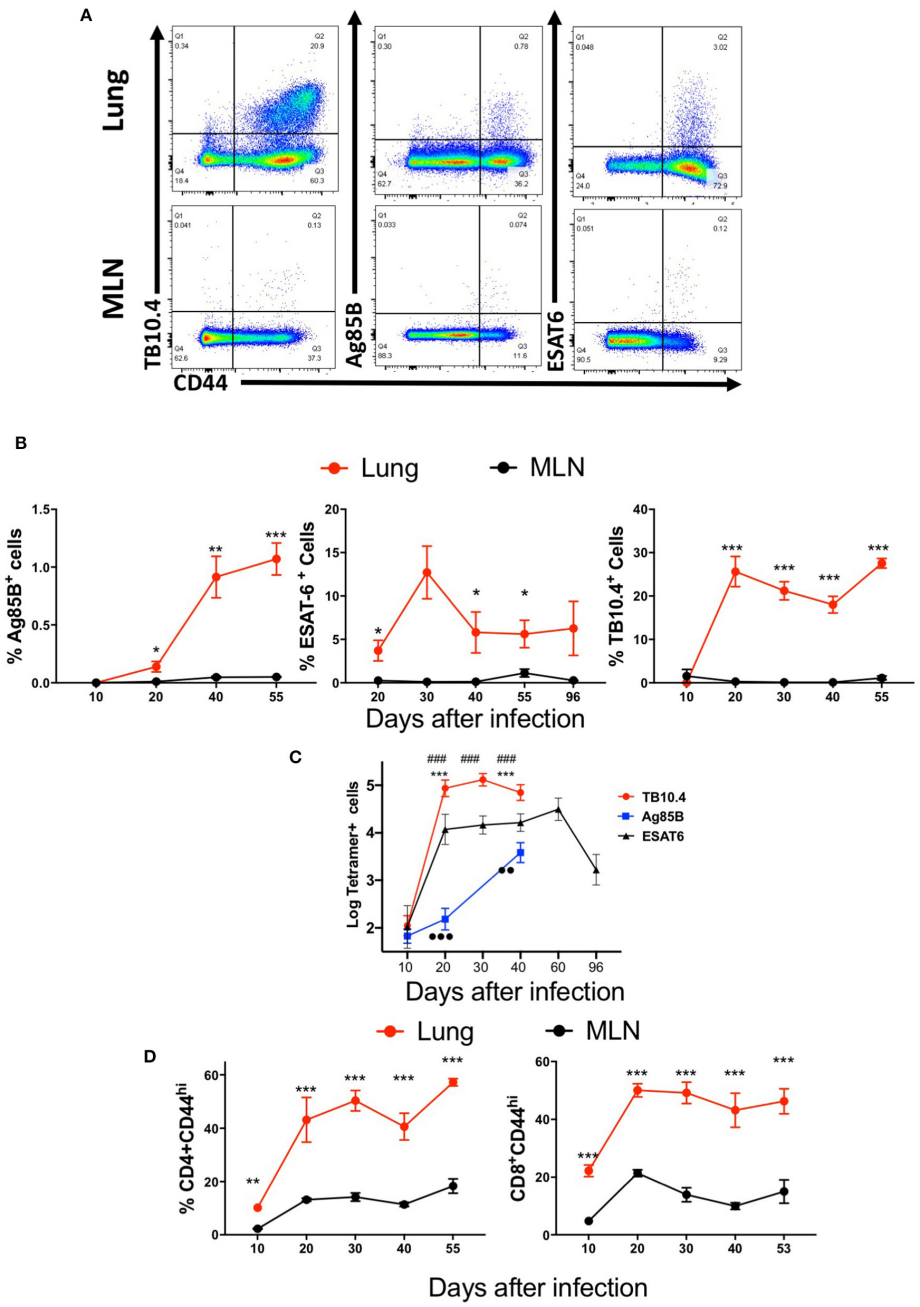
Mycobacteria-specific T cells accumulate in the lung but not the mediastinal lymph node during immunization or infection with *M. tuberculosis*. C57BL/6 mice were infected via aerosol with 250 *M. tuberculosis*, and sacrificed at the indicated days after infection (*n* ≥ 5 per time point). The T cell populations in lung and MLN were analyzed. **(A)** Representative dot plots of tetramer Ag85B, ESAT6, and TB10.4 binding T cells in the lungs and MLN from mice 40 days after infection with *M. tuberculosis* are depicted. **(B)** The mean percentage of tetramer Ag85B, ESAT6, and TB10.4 binding cells gated within the CD44+ CD4 and CD8 T cell populations in the lung or MLN from mice (*n* ≥ 5 per time point) at different days after infection ± SEM are displayed. Differences in frequencies tetramer binding cells between lung and MLN at a given time point after infection are significant at *p ≤ 0.05, **p ≤ 0.01, and ***p ≤ 0.001 (Welch's *t-*test with Holm-Sidak correction for multiple comparisons). **(C)** The mean log_1_0 transformed number of tetramer^+^ Ag85B, ESAT6 and TB10.4 cells ± SD at the indicated days after aerosol infection with *M. tuberculosis*. Differences in cell numbers between *Ag85B and TB10.4; # TB10.4 and ESAT6; and •-Ag85B and ESAT6 are significant at *p* < 0.01 and *p* < 0.001 (2 or 3 symbols) were calculated using Welch's *t* test with Holm-Sidak correction for multiple comparisons. **(D)** The mean frequencies of CD44+ CD4 and CD8 T cells in the lung or MLN from mice at the indicated days after infection with *M. tuberculosis* are shown. Differences are significant at **p ≤ 0.01, and ***p ≤ 0.001 (Welch's *t-*test with Holm-Sidak correction for multiple comparisons).

We first compared the frequency and numbers of mycobacterial Ag85B_240−254_ and ESAT6_1−20_ tetramer-binding CD4 T cells and TB10.4_4−11_-specific CD8 T cells in the lungs and MLN of *M. tuberculosis*-infected mice (Figures 1A–C). TB10.4_4−11_-tetramer-binding CD8 T cells constituted ~1/3 of the total CD44+ CD8 T cells and showed a distinct accumulation early after infection (Figures 1B,C). In comparison, Ag85B-specific CD4 T cells were ~1% of the activated CD4 T cells, with a relatively more delayed accumulation after infection (Figure 1B), while ESAT6 tetramer-binding cells constituted more than 5% of activated CD4 T cells, with a relatively early increase early after infection (Figures 1B,C).

Surprisingly, Ag85B, ESAT6, and TB10.4 tetramer-binding T cells were either not observed or detected at very low frequencies in the MLN of mice at all times after *M. tuberculosis*-infection measured, despite the presence of high frequencies of T cells of the same specificities in the lung (Figures 1A,B).

CD4 and CD8 T cells were activated in the MLN after infection with *M. tuberculosis* as measured by the expression of CD44 as compared to uninfected controls (Figure 1D). However, the frequencies of activated CD4 or CD8 T cells were lower than those in the lungs (Figure 1D).

**Figure 2:**
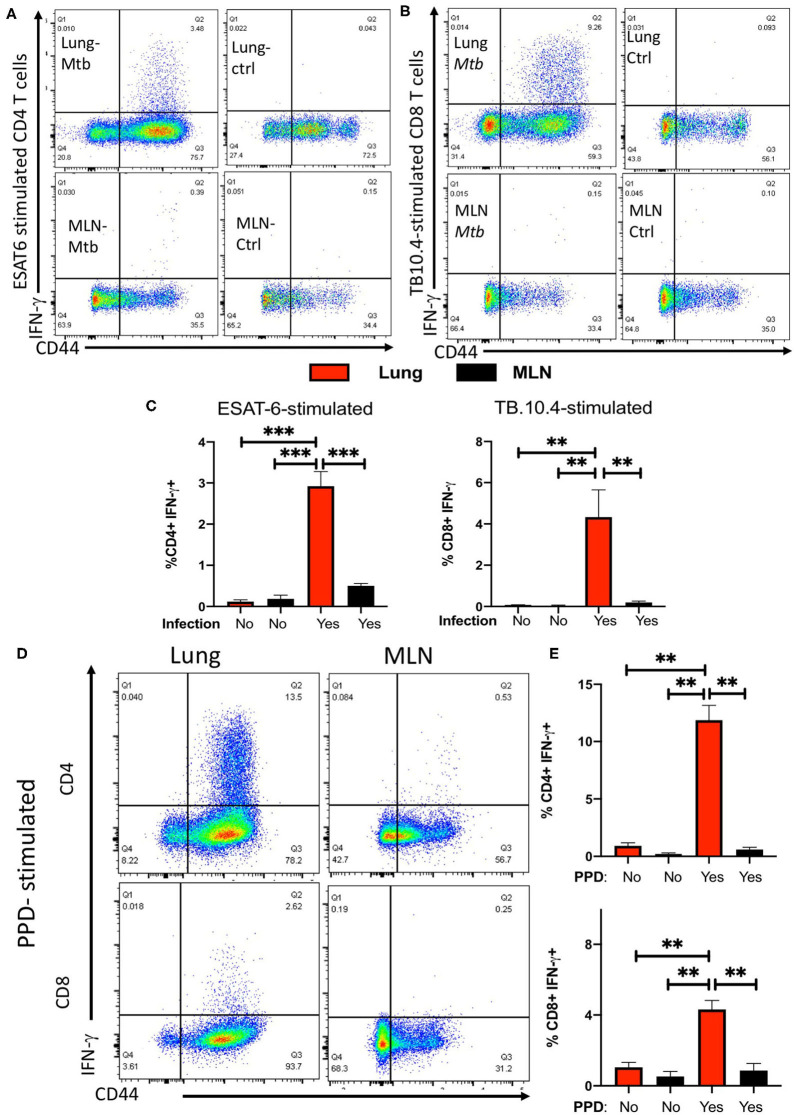
Infection with *M. tuberculosis* results in the accumulation of ESAT-6 and TB10.4-specific IFN-γ-secreting T cells in the lung but not in the MLNs. Representative dot plots showing the CD44+ expression and IFN-γ secretion by lung or MLN CD4 **(A)** or CD8 **(B)** T cells from mice at 4 weeks after infection with *M.tuberculosis* or uninfected controls. Lung and MLN cell suspensions were stimulated with 20 μg/ml of either ESAT6_1−15_ **(A)** or TB10.4_4−11_ **(B)** peptides for 6 hrs. IFN-γ was determined by ICS after 4 h incubation with brefeldin A as described in the materials and methods section. **(C)** The mean frequencies of IFN-γ secretion ± SEM from lung or MLN CD4 and CD8 T cells from mice at 60 days after infection with *M. tuberculosis* or uninfected controls. Lung cell suspensions were stimulated with 10 μg/ml ESAT6_1−15_ or TB10.4_4−11_ peptides for 6 h or were left untreated. Differences of IFN-γ-secreting cell frequencies with stimulated lung cells are significant at **p ≤ 0.01, and ***p ≤ 0.001 (2-way ANOVA). Representative dot plots **(D)** and the mean frequencies **(E)** of IFN-γ-secreting ± SEM lung or MLN CD4 and CD8 T cells from mice 30 days after infection with *M. tuberculosis* stimulated or not with PPD. Differences of between groups are significant at **p ≤ 0.01 (2-way ANOVA).

To further support this observation, the frequencies of IFN-γ-secreting CD4 and CD8 T cells in response to ESAT6_1−15_ and TB10.4_4−11_ peptide stimulation were evaluated in the lung and MLNs of *M. tuberculosis*-infected mice. Lung T cells from mice infected with *M. tuberculosis* secreted IFN-γ after stimulation with the ESAT6 and TB-10.4 peptides, while no IFN-γ responses were determined in T cells from lungs from non-infected mice (Figures 2A–C). CD4, but not CD8 T cells, responded to ESAT-6 peptide stimulation by producing IFN-γ. Instead, CD8 but not CD4 T cells showed substantial IFN-γ responses to TB10.4 peptide stimulation (Supplementary Figure 1A).

In contrast to the response observed by lung CD4 and CD8 T cells, MLN T cells showed low frequencies or undetectable levels of IFN-γ-secreting cells in response to peptide stimulation (Figures 2A–C). CD4 T cells from lungs but not MLNs from *M. tuberculosis*-infected mice secreted IFN-γ in the absence of peptide stimulation (Supplementary Figure 1B). IFN-γ secretion was neither detected in unstimulated lung CD8 T cells from *M. tuberculosis*-infected mice nor in CD4 T cells from uninfected mice (Supplementary Figures 1B,C). CD4 and CD8 T cells from lungs or MLN from infected or control mice secreted IFN-γ in response to PMA/ionomycin stimulation. However, both CD4 and CD8 T cells from lungs from *M. tuberculosis*-infected mice secreted higher levels of IFN-γ as compared to MLN cells from infected mice, or to lung or MLN T cells from uninfected controls (Supplementary Figures 1D–F).

To exclude that T cell specific for other mycobacterial antigens are present in the MLN, we compared the IFN-γ responses by lung and MLN T cells from *M. tuberculosis* infected mice stimulated with PPD were compared. PPD is a purified protein fraction isolated from culture media filtrates of *M. tuberculosis* that contains more than 300 different proteins (22, 24, 25). Lung CD4 and CD8 T cells but not MLN T cells produced IFN-γ in response to PPD stimulation (Figures 2D,E). The levels of IFN-γ secreted by MLN T cells stimulated with PPD were similar to those secreted by non-stimulated controls from infected mice (Figure 2E).

**Figure 3:**
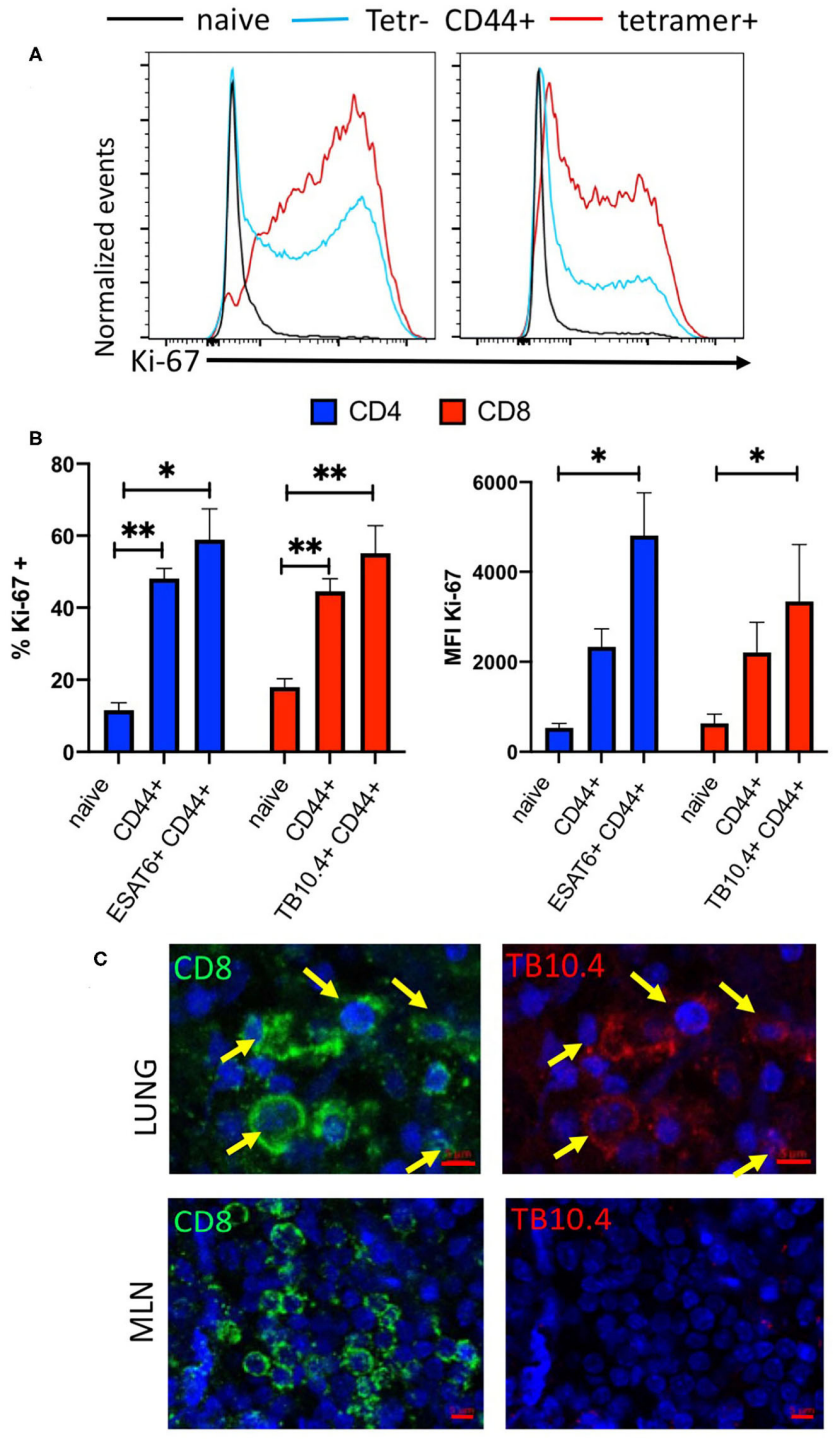
Infection with *M. tuberculosis* results in the accumulation of *M. tuberculosis* specific proliferating T cells in the lung. **(A)** Representative histograms showing Ki67 expression in naïve (CD44 neg), CD44+ tetramer binding (ESAT6 or TB10.4 for CD4 or CD8 T cells, respectively) and non-binding CD44+ CD4 or CD8 T cells from mice at 4 weeks after infection with *M. tuberculosis*. **(B)** The mean frequency of Ki67+ CD4 and CD8 T cells ± SEM from lung of from mice either 4 weeks after infection with *M. tuberculosis*. Total CD44+ CD4 and CD8 T cells as well as tetramer ESAT6 or TB10.4+ CD4 or CD8 T cells are depicted (*n* = 6 per group). The frequencies of Ki67 cells are compared to those of CD44- naïve CD4 or CD8 T cell in the same lung. Differences with lung T cells from naïve cells are significant at *p ≤ 0.05 and **p ≤ 0.01 (one-way ANOVA). **(C)** Double immunolabelling with TB10.4 tetramer and CD8 in lung and MLN slices from mice (*n* = 3) 8 weeks after infection with *M. tuberculosis* was performed as described in the materials and methods section. Representative micrographs are shown.

The frequency and expression levels of the proliferation marker Ki-67 in ESAT6 and TB10.4 tetramer-binding CD4 and CD8 T cells in the lung of *M. tuberculosis* infected mice were increased as compared with those in naïve T cells (Figures 3A,B). The frequency of cells non-tetramer binding activated CD4 and CD8 T cells was also increased compared to that of naïve T cells.

Moreover, the immunostaining of lung slices from *M. tuberculosis* infected mice with TB10.4 tetramer overlapped as expected with CD8 labeled cells and showed a clustered distribution in the lung lesions of infected mice. TB10.4 labeling was not observed in MLN sections of the same animal (Figure 3C, Supplementary Figure 1G).

### Kinetics of Accumulation of Specific T_RM_ Cells in the Lungs of *M. tuberculosis*-Infected Mice

**Figure 4:**
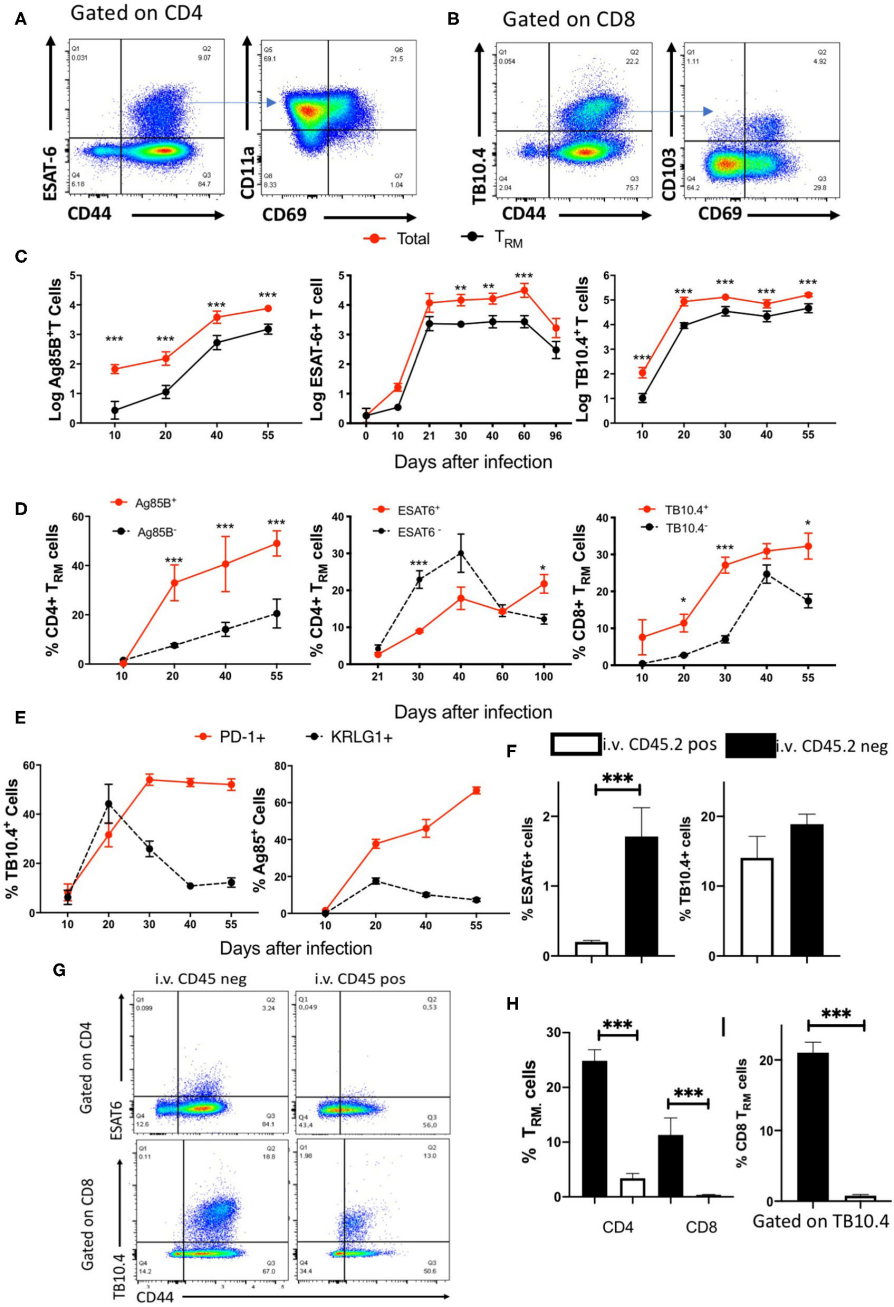
Kinetics of specific TRM cell accumulation in the lungs of *M. tuberculosis*-infected mice. **(A)** Representative dot plots and gating strategy of tetramer ESAT-6 binding CD11a+CD69+ CD4TRM cells in the lungs of mice 4 weeks after *M. tuberculosis* infection. **(B)** Representative dot plots and gating strategy of tetramer TB10.4 binding and CD103+CD69+ CD8 TRM cells in the lungs of mice 4 weeks after *M. tuberculosis* infection. **(C)** The mean log10 of total and TRM tetramer binding Ag85B, ESAT6, and TB10.4 cell numbers in the lungs of mice at different time points after *M. tuberculosis* ± SEM are depicted. Differences between total and TRM cell numbers at each time point are significant (*p ≤ 0.05, **p ≤ 0.05, and ***p ≤ 0.001, Welch's *t-*test with Holm-Sidak correction for multiple comparisons). **(D)** The mean frequencies of lung CD4 or CD8 TRM cells within tetramer positive or negative populations ± SEM at different times after infection with *M. tuberculosis* are shown. The frequencies of TRM are calculated with respect to total CD4 or CD8 CD44+ tetramer positive or negative T cells. Differences are significant at *p ≤ 0.05, **p ≤ 0.05, and ***p ≤ 0.001 calculated using Welch's *t-*test with Holm-Sidak correction for multiple comparisons. **(E)** The mean frequencies of PD1+ and KRLG1+ within Ag85B and TB10.4 tetramer-binding cells in the lung of mice at different times after infection with *M. tuberculosis* ± SEM is shown. Ag85B and TB10.4 tetramer positive cells were gated on CD4 and CD8 cells, respectively. Differences in frequencies of PD1 and KRLG1 positive cell populations are significant at *p ≤ 0.05 and ***p ≤ 0.001(Student's *t-*test with Holm-Sidak correction for multiple comparisons). **(F)** The mean ± SEM (*n* = 6) of i.v. CD45.2 positive or negative ESAT6 and TB10.4 tetramer binding within CD4 and CD8T, respectively, cells in the lung of mice 30 days after infection with *M. tuberculosis* (*n* = 6). Differences are significant at ***p ≤ 0.001 Welch's *t-*test. **(G)** Representative dot plots of i.v. CD45.2 labeled or unlabeled ESAT6 and TB10.4 tetramer binding T cells in the lung of mice 30 days after infection with *M. tuberculosis*. **(H)** The mean frequencies ± SEM (*n* = 6) of i.v. CD45.2 positive or negative CD11a+CD69+ CD4 TRM and CD103+ CD69+ CD8 TRM cells within activated CD4 and CD8 T cells, respectively, in the lung of mice 30 days after infection with *M. tuberculosis*. Differences are significant at ***p ≤ 0.001 Welch's *t-*test. **(I)** The mean ± SEM (*n* = 6) of i.v. CD45.2 positive and i.v. CD45.2 negative TB10.4 tetramer binding CD103+CD69+ CD8 TRM cells in the lung parenchyma and vasculature of mice 30 days after infection with *M. tuberculosis*. Differences are significant at ***p ≤ 0.001 Welch's *t-*test.

The frequency and numbers of pulmonary CD4 and CD8 T_RM_ during *M. tuberculosis* infection were then determined (Figures 4A,B). CD4 T_RM_ were distinguished from circulating T effector-memory cells based on upregulated expression of the early activation marker CD69 and the integrin CD11a (10, 26, 27), whereas CD8 T_RM_ were characterized by the expression of the αE integrin CD103 and CD69 (10). Lung T_RM_ were detected at all time points after 20 days of *M. tuberculosis* infection. While the number of mycobacteria-specific T_RM_ followed that of the total mycobacteria-specific T cells (Figure 3C), the frequencies and numbers of T_RM_ within the antigen-specific and total T cells showed important differences (Figure 4D, Supplementary Figures 2A,B). The frequencies of Ag85B and TB10.4-specific T_RM_ cells were higher than those of T_RM_ within tetramer-negative T cells. Instead, the frequency of ESAT6 tetramer-binding CD4 T_RM_ cells was lower than in the tetramer-negative population (Figure 4D). TB10.4 tetramer-binding T_RM_ constituted ca 50% of the total CD8 T_RM_ cell numbers in the infected lungs (Supplementary Figures 2A,B).

In order to further characterize the antigen-specific T cells induced locally in the lung during infection with *M. tuberculosis*, we measured the expression of the PD-1 and KLRG1. PD-1+ CD4 T cells have been shown to display proliferative and protective capacity and residence in the lung parenchyma after *M. tuberculosis* infection, while KLRG1 associated with short-lived terminally differentiated effector cells (28–30). The expression of KRLG1 in mycobacteria-specific T cells peaked at 3 weeks after infection and decreased thereafter (Figure 3E). Most T_RM_ cells in the lung did not express KRLG1 (Supplementary Figure 2C). The frequency of PD1+ Ag85B and TB10.4-specific CD4 and CD8 T cells increased during infection with *M. tuberculosis* and remained elevated at the late time points of infection (Figure 4E).

In order to confirm that CD8 and CD4 T_RM_ are bona fide parenchymal resident cells we performed intravascular labeling with anti-CD45.2 mAb. Mice were sacrificed 3–5 min after inoculation. Lymph nodes leukocytes remained unlabeled, all blood leukocytes were labeled by anti CD45.2 and ~50% of lung leukocytes were labeled (Supplementary Figure 2D and not shown). Approximately 70% of CD4 and CD8 T cells from mice 30 days after infection with *M. tuberculosis* resided in the lung parenchyma (Figure 2E). While most tetramer ESAT6 binding CD4 T cells located in the parenchyma while TB10.4 tetramer labeled cells localized both in the vasculature and the parenchyma, indicating a skewed distribution of the mycobacterial specific T cells (Figures 4F,G). As expected, both CD4 and CD8 T_RM_ (total and tetramer TB10.4 binding cells) located in the lung parenchyma (Figures 4H,I, Supplementary Figures 2F,G). Thus, CD8 and CD4 T_RM_ are bona fide parenchymal resident cells.

### Mycobacteria-Specific T Cells Accumulate in the Lung but Not the Mediastinal Lymph Node After Mucosal BCG Immunization

We then compared the kinetics of generation of mycobacteria-specific T cells in the lung and the MLN of mice 3 weeks after either i.t. or s.c. BCG immunization.

**Figure 5:**
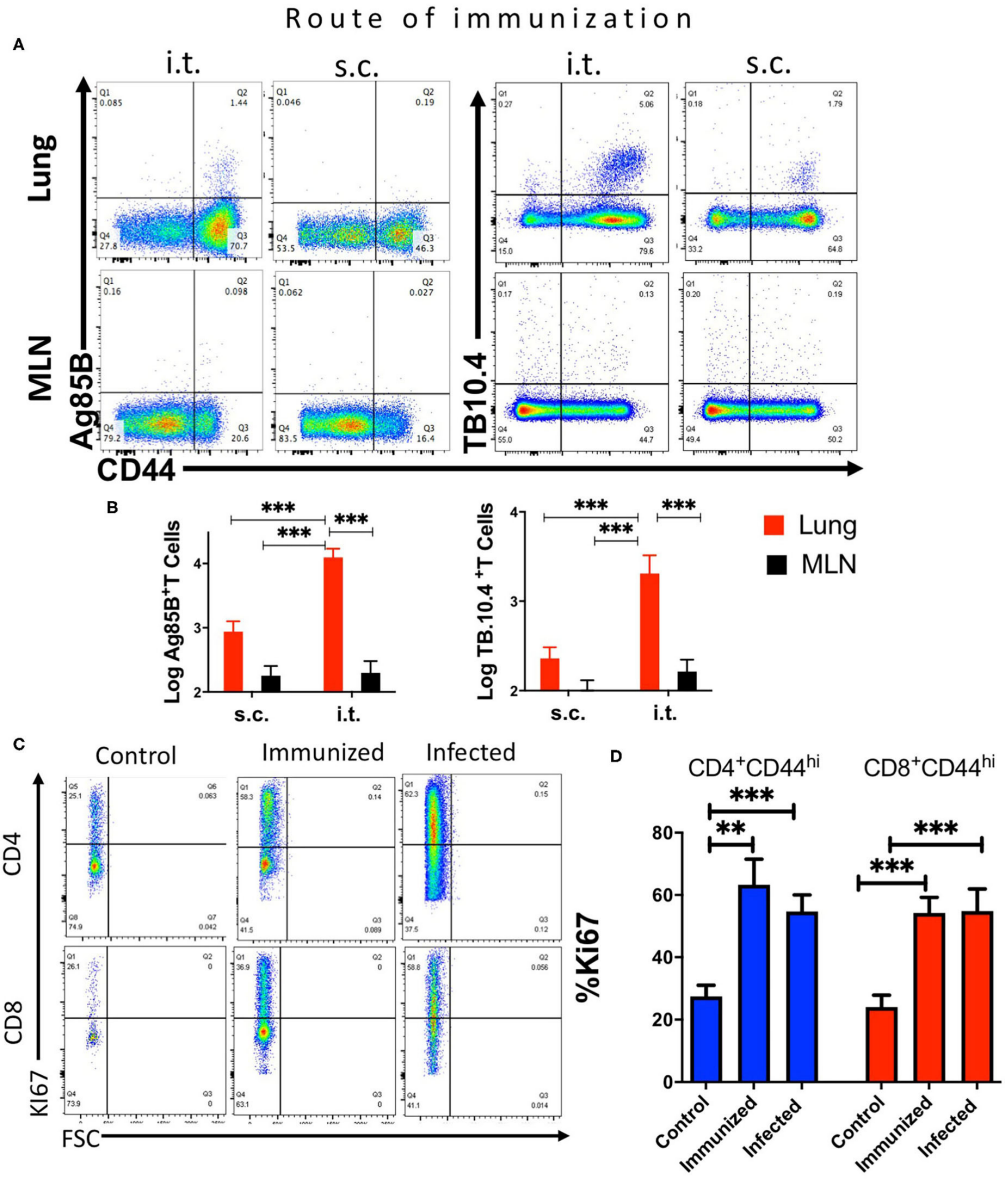
Mycobacteria-specific T cells accumulate in the lung but not the mediastinal lymph node during immunization or infection with *M. tuberculosis*. C57BL/6 mice were immunized i.t. or s.c. with 107 BCG and T cell populations analyzed in the lung and MLN cell suspensions 3 weeks after immunization. **(A)** Representative dot plots of tetramer Ag85B and TB10.4 binding CD4 or CD8 T cells, respectively, in lungs and MLN from mice 3 weeks after i.t. or s.c. BCG immunization are shown. **(B)** The mean percentage of tetramer Ag85B and TB10.4 binding cells gated within the CD44+ CD4 and CD8 T cell populations in the lung or MLN from BCG-immunized mice. Differences in frequencies tetramer binding cells between lung and MLN at a given time point after infection are significant at **p ≤ 0.01, and ***p ≤ 0.001(Welch's *t-*test with Holm-Sidak correction for multiple comparisons). **(C)** Representative dot plots showing the frequency of Ki67+ gated in CD44+ CD4 or CD8 T cells in the lung from mice either 4 weeks after infection with *M. tuberculosis*, 3 weeks after i.t. BCG immunization or left untreated are shown. **(D)** The mean frequency of Ki67+ CD4 and CD8 T cells ± SEM from lung of mice 4 weeks after infection with *M. tuberculosis*, 3 weeks after BCG immunization and non-immunized control mice (*n* ≥ 5 per group) are shown. Differences with lung T cells from uninfected mice are significant at **p ≤ 0.01 and ***p ≤ 0.001 (Welch's *t-*test with Holm-Sidak correction for multiple comparisons).

We found that the i.t. immunization induced a higher number of mycobacterial Ag85B_240−254_ and TB10.4_4−11_ tetramer- binding CD4 and CD8 T cells in the lung as compared to those immunized via the s.c. route (Figures 5A,B). Instead, Ag85B or TB10.4 tetramer-binding CD4 or CD8 T cells in the MLN were undetectable or had very low numbers after either i.t. or s.c. BCG immunization (Figures 5A,B).

The frequency of Ki-67 activated CD44+ CD4 and CD8 T cells increased in the lungs of i.t. BCG-immunized mice as compared to that of naïve mice, indicating that T cells proliferated in the lung in both BCG-immunized as well as in *M. tuberculosis*-infected mice (Figures 5C,D). Thus, the results suggest that after BCG immunization or *M. tuberculosis* infection specific T cells are generated in the lungs.

**Figure 6:**
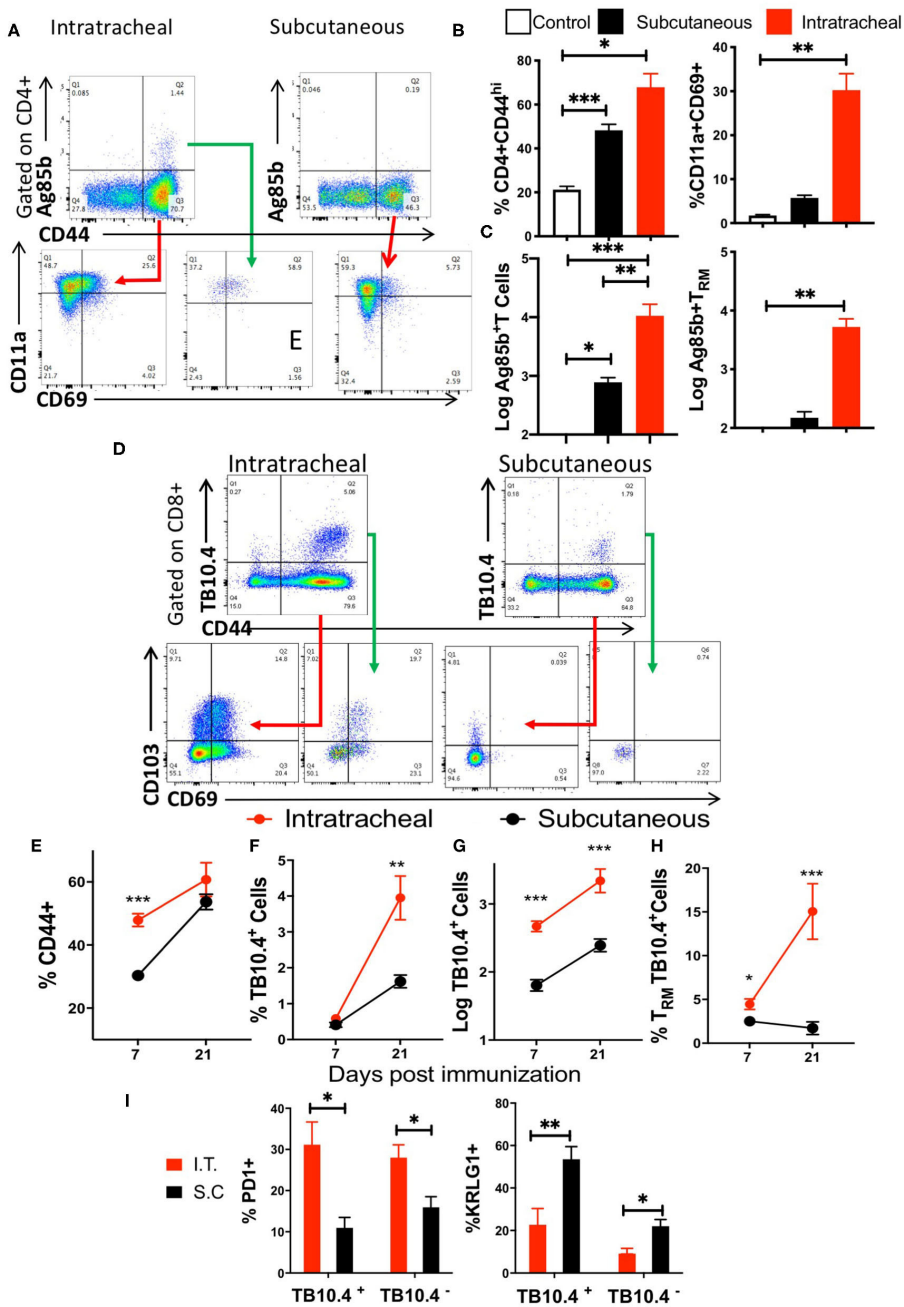
Mycobacteria-specific Trm cells accumulate in the lung during BCG immunization. **(A)** Representative dot plots and gating strategy of tetramer Ag85B binding and CD11a+CD69+ CD4TRM cells in the lungs of mice 3 weeks after i.t. and s.c. BCG immunization. **(B)** Mice were immunized with BCG s.c. or i.t. and sacrificed 3 weeks later. A group of mice was left untreated. The frequency of CD44+ in gated CD3+ CD4 T cells from lungs and of CD4 TRM ± SEM gated on CD3+ CD44+ cells are shown. The mean % ± SEM of at least 4 animals per group are shown. Differences between groups are significant at *p ≤ 0.05 and **p ≤ 0.001 (Welch's *t-*test with Holm-Sidak correction for multiple comparisons). **(C)** The mean numbers ± SEM of tetramer Ag85B binding total and TRM CD4 T cells in the lungs of i.t. and s.c. BCG immunized mice are shown (*n* ≥ 5 animals per group). Differences between groups are significant at **p ≤ 0.001 and ***p ≤ 0.001 (one-way ANOVA). **(D)** Mice were immunized i.t. or s.c. with BCG and sacrificed 1 or 3 weeks later. The representative dot plots show the presence of tetramer TB10.4 binding CD44+ CD8 T cells in the lungs of i.t. or s.c. BCG-immunized mice. The CD69+CD103+ CD8 TRM within the tetramer TB10.4 positive or negative cells are also shown. **(E–H)** The frequency of CD44+ gated on CD3+ CD8 T cells **(E)**, of tetramer TB10.4+ within CD44+ CD8 T cells **(F)**, the numbers of tetramer TB10.4+ CD8 T cells **(G)** and the frequency of TRM cells within all TB10.4 tetramer binding cells **(H)** were determined in lungs from mice at 1 and 3 weeks after i.t. or s.c. immunization with BCG. The mean frequencies or log10 transformed cell numbers ± SEM (*n* = 5 per group) are shown. Differences between groups were statistically significant at *p ≤ 0.05, **p ≤ 0.001, and ***p ≤ 0.001 (Welch's *t-*test with Holm-Sidak correction for multiple comparisons). **(I)** The mean frequencies of PD1+ and KRLG1+ cells within the TB10.4 tetramer + or—CD44+ CD8 T cells in the lungs of mice immunized i.t. or s.c. with BCG 3 weeks before sacrifice are shown. Differences between i.t. and s.c. immunized mice (*n* = 5 per group) were significant at *p ≤ 0.05 and **p ≤ 0.01 (Welch's *t-*test with Holm-Sidak correction for multiple comparisons).

The levels of lung T_RM_ after i.t. and s.c. BCG immunization were then evaluated (Figure 6A). We found increased frequencies of CD44+ CD4 T and CD4 T_RM_ cells in lungs from mice after i.t. as compared to s.c. BCG immunization (Figure 6B). CD4 T_RM_ were observed at 14 but not 7 days after i.t. immunization, and remained at high levels 45 days after BCG administration, while CD4T_RM_ were undetectable at different time points after s.c. immunization (Supplementary Figure 3A). Ten-fold higher numbers of Ag85B tetramer-binding cells were present in the lungs of i.t. immunized compared to those immunized s.c. BCG (Figure 6C). Ag85B-specific lung CD4 T_RM_ in mice immunized s.c. were undetectable (Figure 6C).

We then compared the accumulation of TB10.4_4−11_ tetramer-binding CD8 T cells in the lungs of mice 1 and 3 weeks after i.t. or s.c. BCG immunization (Figure 6D). The frequency of activated CD8 T cells and the number and frequency of TB10.4 tetramer-binding CD8 cells increased from 1 to 3 weeks after immunization (Figures 6E–G). The frequency of tetramer-binding TB10.4 T_RM_ within the lung CD8 T cells increased between 1 and 3 weeks after i.t. BCG immunization (Figure 6F). TB10.4-specific CD8 T cell were detected already 1 week after immunization and their numbers were higher in i.t. than in s.c. BCG-immunized mice (Figure 6G). T_RM_ were not detected within the TB10.4-specific CD8 T cells in the lungs of s.c. immunized mice, while their frequency increased 3 weeks after i.t. BCG immunization as compared to those detected 1 week after immunization (Figures 6D,H).

To further characterize the antigen-specific lung T cells following i.t. and s.c. BCG immunization PD1 and KRLG1 expression were assessed (Supplementary Figure 3B). We found that 30% of mycobacteria-specific CD8 T cells in the lung of mice immunized i.t. with BCG expressed PD1. In comparison, the percentage of PD1-expressing cells was reduced in TB10.4 tetramer-binding and in total CD8 T cells in the lungs of s.c. immunized mice (Figure 6I). In contrast, the frequency of KRLG1+ cells was increased in lungs of mice immunized via the s.c. route as compared to those obtained from i.t. vaccinated mice (Figure 5I).

### Bona Fide T_RM_ Develop in the Lungs of Mice After Mucosal BCG Immunization

**Figure 7:**
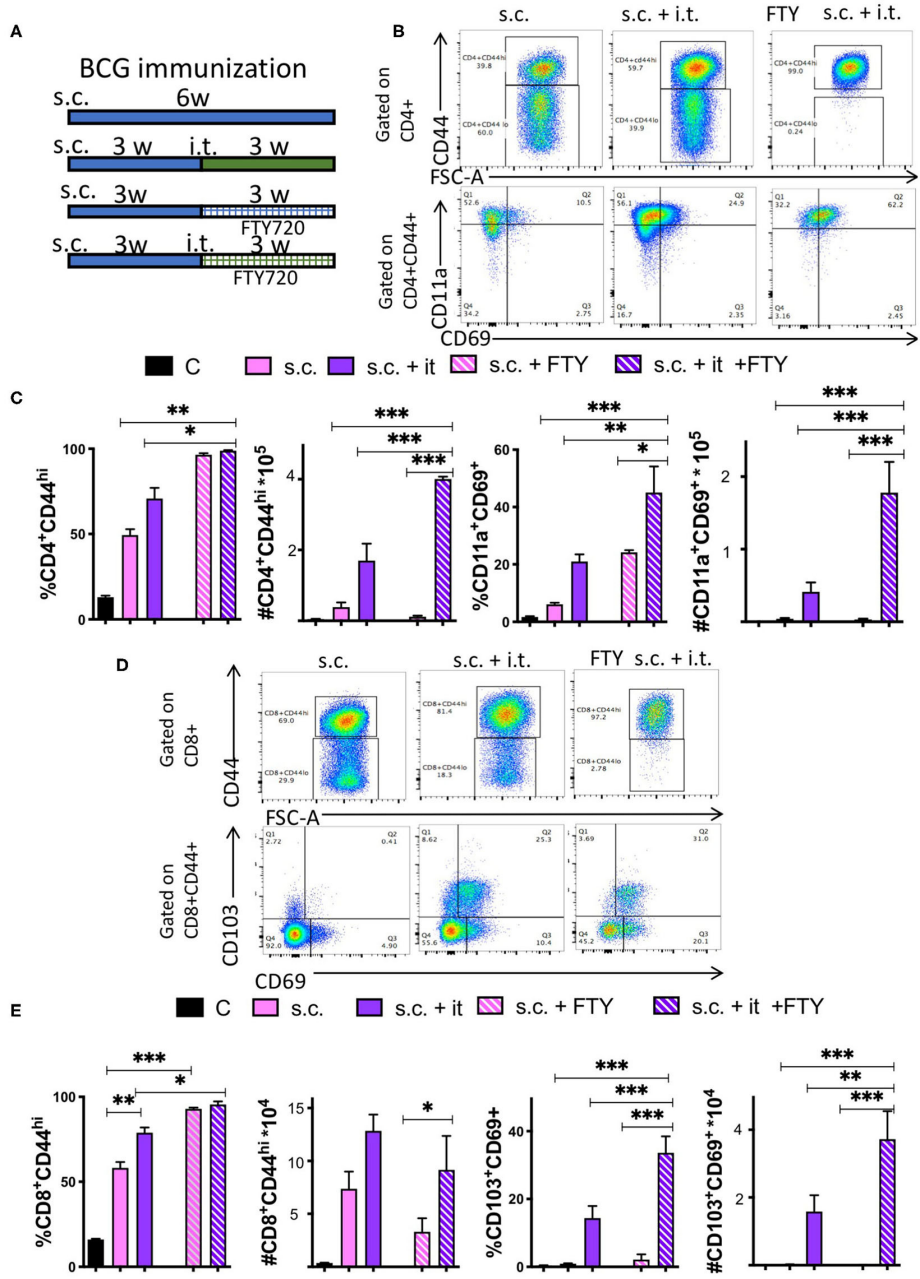
Bona fide TRM develop in the lungs of mice after mucosal BCG immunization. **(A)** Mice were immunized s.c. with 107 BCG and revaccinated or not i.t. with BCG 3 weeks after. Group of immunized and revaccinated mice were treated with 1 mg/kg i.p. FTY320 daily starting 1 day after revaccination until sacrifice. Mice were sacrificed 3 weeks after revaccination. Representative dot plots **(B)** and the mean frequencies and cell numbers **(C)** of pulmonary CD4+CD44+ and of CD11a+CD69+ TRM ± SEM (*n* ≥ 5) in the lung of mice are shown. Differences between experimental groups are significant at *p ≤ 0.05, **p ≤ 0.01, and ***p ≤ 0.001 (one way ANOVA). **(D,E)** Mice were immunized with BCG and treated with FTY720 as described in A-B and Supplementary Figure 2. Representative dot plots **(D)** and the mean frequencies and numbers **(E)** of pulmonary CD8+CD44+ T cells and of CD103+CD69+ TRM in the lungs of mice 3 weeks after revaccination ± SEM (*n* ≥ 5) are shown. Differences between groups are significant at *p ≤ 0.05, **p ≤ 0.01, and ***p ≤ 0.001 (one way ANOVA).

We used the S1PR agonist FTY720 (fingolimod) to block egress of T cells from the lungs or lymph nodes, in order to assess whether T cells could be generated in the mice during BCG immunization. All groups of mice were vaccinated s.c. with BCG and after 3 weeks mice were treated daily i.p. with 1 mg/kg FTY720. One day after the first FTY720 administration, mice were boosted i.t. with BCG (Figure 7A). The administration of FTY720 reduced by more than 90% the number of blood T cells already 15 h after inoculation of a single dose (Supplementary Figure 4A). The i.t. booster increased protection of mice against challenge with *M. tuberculosis* as shown by the reduced levels of bacteria in lungs and spleens (Supplementary Figures 4B,C).

The frequency of CD44+ CD4 and CD8 T cells in lungs increased in both groups (boosted or not) after FTY720 treatment due to the fall of naïve T cells (Figures 7B–E). The number of CD44+ CD4 T cells in the lung of i.t. BCG-boosted animals increased after FTY720 administration (Figure 7C), while both CD44+ CD4 and CD8 T cell numbers were reduced in the s.c. immunized group as compared to those not treated with FTY720. Moreover, the numbers and frequencies of CD4 and CD8 T_RM_ increased in i.t. BCG re-vaccinated mice treated with FTY720, while numbers of CD4 T_RM_ in s.c. BCG immunized but not re-vaccinated mice remained very low, been mostly undetectable (Figures 7B–E).

Since inoculation of FTY720 was done before i.t. immunization, our results indicate that T_RM_ generation and accumulation in the lung takes place in the absence of lymphoid recirculation after i.t. immunization, suggesting a minor role for MLN if any in this process.

## Discussion

Our data indicate that aerosol infection with *M. tuberculosis* and mucosal immunization with BCG results in the generation, proliferation and persistence of specific T cells (a fraction exhibiting T_RM_ markers) in the lung, independently of the recirculation from the draining lymph nodes.

It is generally accepted that following antigen encounter in the lung, during infection or immunization dendritic cells (DCs) will migrate to the MLNs and present mycobacterial antigens and different co-stimulatory signals to specific naïve T-cells. The low frequency of naïve T cells specific for any one pathogen epitope means dependence on primary responses initiated in draining lymph nodes, often allowing time for a serious infection to develop. The activated T cells will proliferate and differentiate into effector T-cells and memory T-cells that are distributed more broadly throughout the body. Upon re-exposure to the pathogen, memory T-cells are able to mount a more rapid and robust antigen-specific responses (10, 31).

Our results argue that during infection with *M. tuberculosis* or i.t. BCG immunization, mycobacteria*-*specific T cells are generated in the lung. The absence or low numbers of tetramer-binding *M. tuberculosis*-specific T cells at all time points studied, the low frequencies or absence IFN- γ-secreting cells in the MLNs of infected mice in response to specific peptides well as to a PPD (containing several *M. tuberculosis* proteins), and the expression of the proliferation marker Ki67 in specific CD4 and CD8 T cells from the lung of *M. tuberculosis*-infected or BCG-immunized mice support this possibility.

Although i.t. aerosol immunization is not applicable for human vaccination for practical reasons, it allows for a more accurate delivery of defined doses than with intranasal or aerosol vaccination. While doses of 5 × 10^5^ BCG CFU i.t. have been shown improved protection of mice against *M. tuberculosis* challenge as compared to the s.c. route (4), the 10^7^ BCG dose was chosen for our studies since it has been shown to confer the largest reduction of lung *M. tuberculosis* levels, when compared to lower doses (32). The i.t. immunization with BCG generated higher mycobacteria-specific lung CD4 and CD8 T cells as compared to the s.c. route of delivery. After i.t. BCG immunization or booster an important fraction of total or antigen-specific CD4 and CD8 lung T cells displayed a T_RM_ phenotype. Instead, T_RM_ were low or absent after s.c. BCG administration, as also shown previously in the lung or bronchoalveolar fluid lavage cells (4, 33). Although most experiments were done in mice sacrificed at 3 weeks after immunization, T_RM_ were measured from 14 to 45 but not 7 days after BCG i.t. immunization, but were under detection level in s.c. immunized mice.

KRLG1+ T cells are short-lived terminally differentiated T cells (28). Upon infection, *M. tuberculosis*-specific CD4 T cells expressing KLRG1 exhibited a heightened capacity to secrete IFN-γ (29). PD-1+ T cells secreted less inflammatory cytokines than KLRG1+ counterparts upon re-stimulation, proliferated, showed a higher survival rate and the capacity to differentiate into KLRG1+ cells (29). PD-1+ CD4 T cells mediated protection against *M. tuberculosis*, and PD-1+ T cells and T_RM_ locate in the lung parenchyma as shown here and elsewhere (28, 33). Here we showed that levels of *M. tuberculosis*-specific KRLG1+ CD4 and CD8 T cells were high early (20 dpi) after infection and decreased thereafter, while the levels of PD1+ T cells increased during the first weeks of infection, remaining high at later time points. Most KRLG1+ cells did not express T_RM_ markers.

The i.t. BCG immunization generated higher levels of total or *M. tuberculosis*-specific PD1+ CD4 and CD8 T cells as compared to those generated after s.c. immunization, which showed higher frequency of KRLG1 expression. In line with this, we confirm previous studies showing that mucosal immunization or boosting with BCG or mycobacterial antigens improve vaccine activity compared to the commonly used intradermal BCG (4, 6–8, 32, 34).

T cells are restricted to using the S1P pathway to exit secondary lymphoid organs and enter the blood (35, 36). FTY720 is a S1P receptor modulator that impairs lymphocyte egress from the lymph nodes and other secondary organs (37). Previous studies have shown that once priming has occurred, recruitment from circulation is not needed, implying the protective T-cells locate in the lung (38). Here, FTY720 inoculation indicated that persistent T_RM_ were detected in lungs. The results also suggest that after i.t. immunization with BCG, T_RM_ are generated in the lung independently of recruitment of T cells from lymphoid stores since FTY720 was inoculated before the revaccination.

Immune responses to a variety of antigens have been shown to be initiated directly in the lung by using lymphotoxin (LT)-deficient mice that lack conventional secondary lymphoid organs (39, 40). By means of LT-deficient and splenectomized mice, it was shown that CD8+ T cells could be primed, proliferated, acquired a memory phenotype and cleared a challenge viral infection in the complete absence of secondary lymphoid organs (41). LT-deficient mice were also shown to generate protective T cell responses against *M. tuberculosis* (42–44). In lymph node deficient splenectomized mice infected with influenza, immune responses were initiated without delay (40). Our results indicate that T cells are generated in the lung, excluding functional compensations occurring in genetically deficient mice. We fall short of showing that T cell priming occurs in the lung of infected mice.

Leukocytes that infiltrate the lung have been shown to assemble into inducible bronchus-associated lymphoid tissue (iBALT) after inflammation or infection (45). Like conventional lymphoid organs, iBALT contains segregated B and T cell areas, specialized stromal cells, high endothelial venules, and lymphatic vessels (46). iBALTs might promote encounters between naive lymphocytes recruited from the blood and antigen-presenting cells that have migrated from the lumen of the airways. Naïve T cells were primed within the iBALT (47), and T cells in the iBALT have been suggested to participate in the protective secondary immune responses against pathogens (34, 48).

The pulmonary delivery of *M. tuberculosis* antigen-primed DCs has been shown to led to increased and rapid iBALT formation and improved disease outcome (49). B cell follicles are often observed in *M. tuberculosis* granulomas in mice (50), humans, and monkeys (51). Monkeys with latent *M. tuberculosis* infection maintain large, well-organized areas of iBALTs surrounding granulomas, whereas NHPs with active disease have fewer and less organized areas of iBALT (51).

Different studies have suggested that the antigen-specific T cell responses during *M. tuberculosis* infection show a delayed onset as compared to infection with other pathogens (52–55). These studies used transgenic or retrogenic specific T cell transfers to show T cell activation in the lymph nodes during *M. tuberculosis* infection (54–57). Our data studying suggest that T cell priming or activation in the draining lymph node is not required to generate antigen-specific T cell responses in the lungs. Whether a delay in T cell priming might be instead due to the process of formation of the iBALTs induced by *M. tuberculosis* infection or mucosal vaccination deserves further investigation. We showed qualitatively differences in the mucosal as compared to a distal route of immunization, supporting the use of mucosal immunization for achieving superior immune protection against infection with *M. tuberculosis*.

## Data Availability Statement

The datasets presented in this article are not readily available because all raw data from this article can be obtained upon request to the corresponding author. Requests to access the datasets should be directed to Martin E. Rottenberg, martin.rottenberg@ki.se.

## Ethics Statement

The animal study was reviewed and approved by Stockholm North Ethical Committee.

## Author Contributions

JB, RL, WM, and BC performed the investigation. BC and MR wrote the original draft and reviewed the manuscript. MR conceived the study. All authors approved the final version of this manuscript to be published.

## Conflict of Interest

The authors declare that the research was conducted in the absence of any commercial or financial relationships that could be construed as a potential conflict of interest.
