# Supplementary material for: Mycobacteria-Specific T Cells Are Generated in the Lung During Mucosal BCG Immunization or Infection With Mycobacterium tuberculosis
Source: Front Immunol. 2020 Oct 22;11:566319. doi: 10.3389/fimmu.2020.566319 (PMC7643023; doi:10.3389/fimmu.2020.566319)
Supplement: Supplementary file 1 [file Data_Sheet_1.docx]

Supplementary Figures

## Supplementary Figure 1
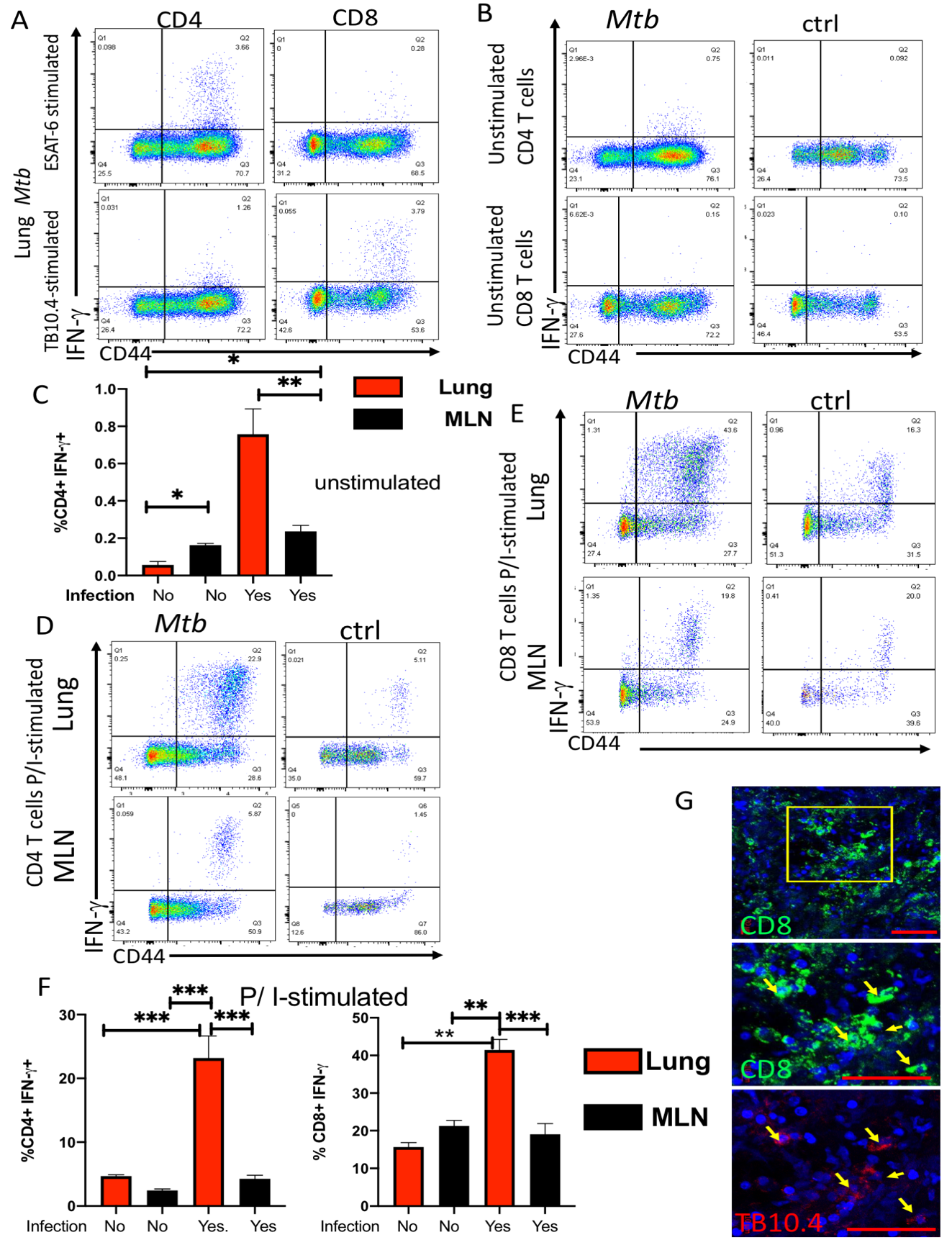


### Mycobacteria-specific T cells accumulate in the lung but not the mediastinal lymph node during BCG immunization and infection with M. tuberculosis

**A**)Representative dot plots showing the CD44 expression and IFN-γ secretion by lung CD4 and CD8 T cells from mice 4 weeks after infection with *M. tuberculosis* or uninfected controls. Lung cell suspensions were stimulated with 10 μg/ml TB10.4 _4-11_ or ESAT6 _1-15_ peptides for 6 hrs. IFN-γ was determined by ICS after 4hrs incubation with brefeldin as described in the materials and methods section. **B**) Representative dot plots showing the CD44 expression and IFN-γ secretion by lung CD4 and CD8 T cells from mice at 4 weeks after infection with *M. tuberculosis* or uninfected controls. Whole lung cell suspensions were incubated with brefeldin during 4 hs and IFN-γ was determined by ICS as described in the materials and methods section. **C**) The mean frequencies ± SEM of IFN-γ secreting CD4 T cells from unstimulated lung or MLN cells from mice at 4 weeks after infection with *M. tuberculosis* or uninfected controls (n≥6). Differences between groups are significant at p≤ 0.05 and p≤0.01 2-way ANOVA. D) Representative dot plots showing the CD44 expression and IFN-γ secretion by lung CD4 T cells from mice 4 weeks after infection with *M. tuberculosis* or uninfected controls. Whole lung cell suspensions were incubated with PMA/ ionomycin and brefeldin and IFN-γ was determined after ICS. **E**) Representative dot plots showing the CD44 expression and IFN-γ secretion by lung CD8 T cells from mice at 4 weeks after infection with *M. tuberculosis* or uninfected controls. Lung cell suspensions were incubated with PMA/ ionomycin and brefeldin for 6 hs and IFN-γ was determined by ICS. **F**) The mean frequencies ± SEM of IFN-γ secreting CD4 and CD8 T cells from lung or MLN cells from mice at 4 weeks after infection with *M. tuberculosis* or uninfected controls (n≥6). Lung cell suspensions were stimulated with PMA and ionomycin for 6 hrs or were left untreated. Differences of IFN-γ-secreting cell frequencies with stimulated lung cells are significant at *p≤0.05, **p≤0.01 and ***p≤0.001 (2-way ANOVA). **G**) Double immunolabelling with TB10.4 tetramer and CD8 in lung slices from mice 8 weeks after infection with *M. tuberculosis* was performed as described in the materials and methods section. A representative micrograph is shown. The arrows show the colocalization of CD8 and the tetramer labelling.

## Supplementary figure 2


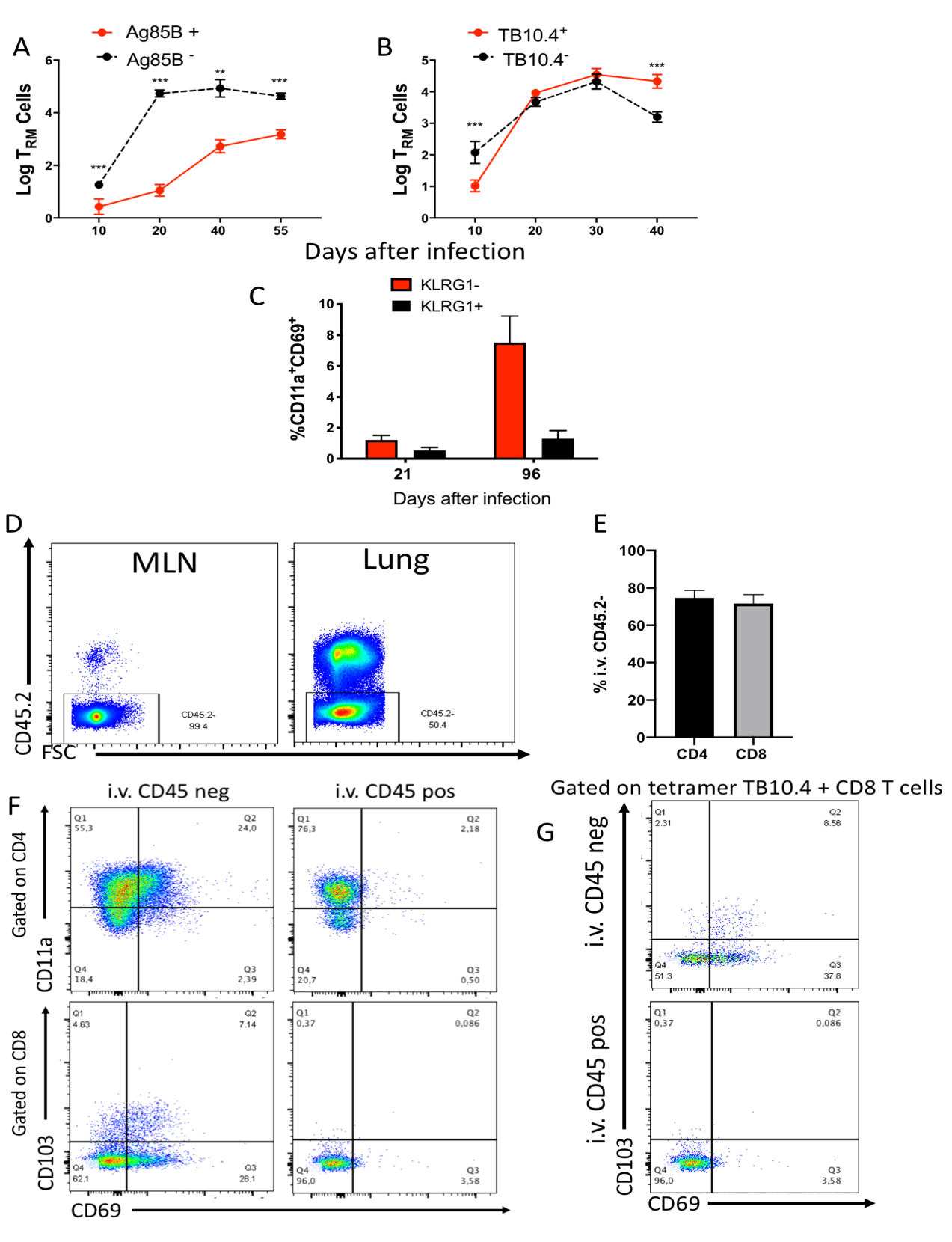


### Distinct kinetics of M. tuberculosis-specific T_RM_ cell accumulation in the lungs of infected mice

The mean log tetramer Ag85B+ and non-binding CD4 T_RM_ CD4 (**A**) and TB10.4+ and TB10.4- CD8 T_RM_ (**B**) in the lung of mice (n ≥ 5 per time point) ± SEM at different times after infection with *M. tuberculosis* is shown. Differences between experimental groups are significant at *p≤0.05, **p≤0.01 and *** p≤0.001 (one way-ANOVA). **C**) he mean frequency of KRLG1- and KRLG1+ CD11a+CD69+ within CD44+ CD4 T cells in lungs from *M. tuberculosis* infected mice is depicted. **D**) Representative dot plots of the CD45.2 labelling on leukocytes (lymphocyte gate) from the lung and MLN from mice 30 days after M. tuberculosis infection sacrificed after in vivo i.v. CD45.2 innoculation. **E**) The mean frequency ± SEM of i.v. CD45.2 negative CD4 and CD8 T cells in the lung from mice(n=6) 30 days after infection with *M. tuberculosis*. **F**) Representative dot plots of i.v. CD45.2 positive or negative gated, CD11a+CD69+ CD4+ and CD103+ CD69+CD8 T_RM_ cells in the lung of mice 30 days after infection with *M. tuberculosis*. **G**) Dot plots of CD103+CD69+ CD8 T_RM_ cells in the lung parenchyma and vasculature of mice 30 days after infection with *M. tuberculosis* gated on TB10.4 tetramer binding cells.

## Supplementary figure 3

###
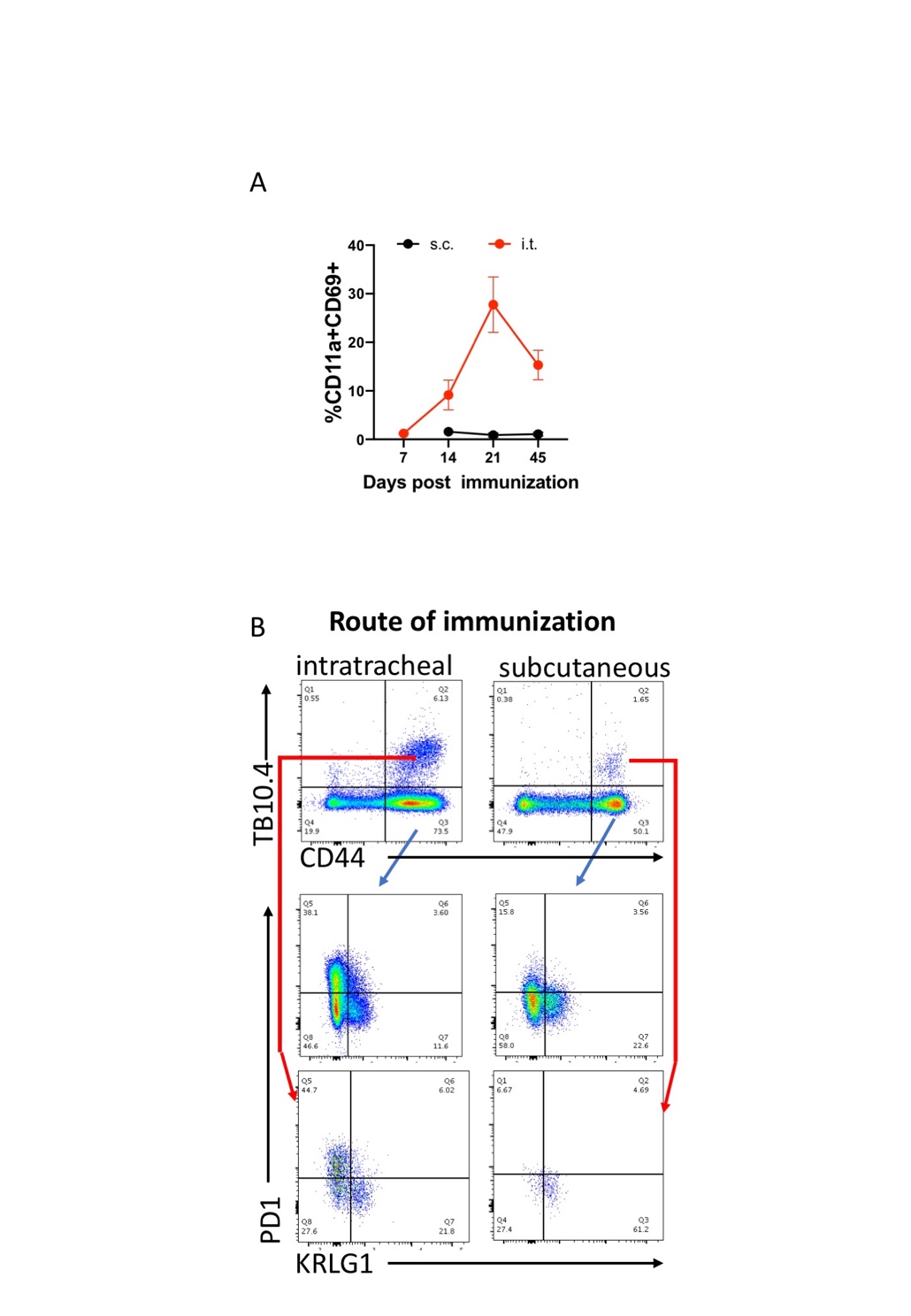


### Intratracheal vaccination increases augments the accumulation CD4+ and CD8+ T_RM_ cells in the lung

**A**) Mice were immunized i.t. or s.c. with 10^7^ BCG and sacrificed at the indicated time points after infection. The frequency of CD4 T_RM_ ± SEM at different times after s.c. or i.t. BCG immunization is depicted (n≥3). **B**) Representative dot plots show the presence of TB10.4 tetramer-binding and total CD44+ CD8 T cells in the lungs of i.t. or s.c. BCG immunized mice. The dot plots also show the presence of PD1+ and KLRG1 within the tetramer TB10.4+ or TB10.4- cells.

## Supplementary figure 4


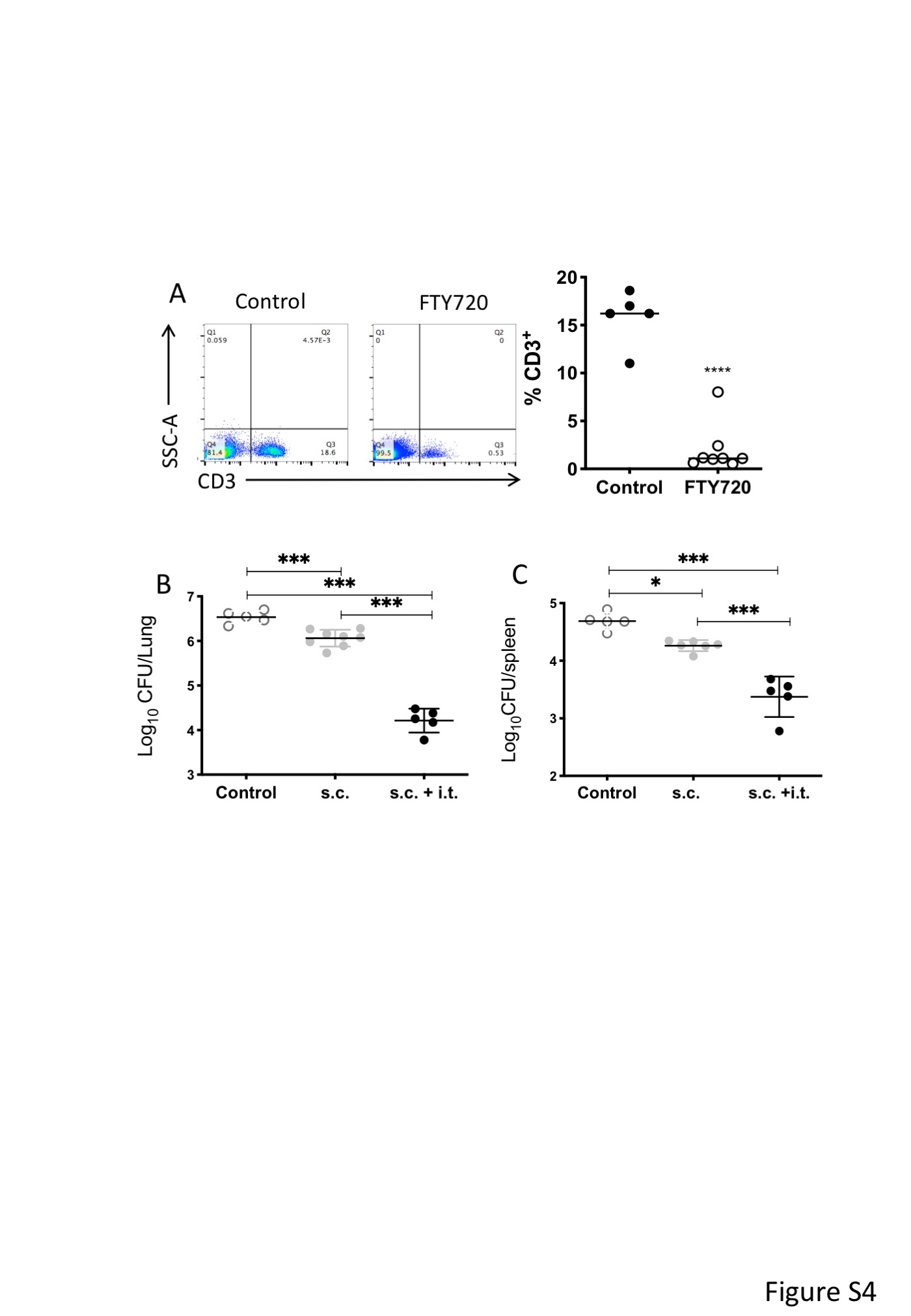


### T cells are generated in the lungs of BCG-immunized mice via the intratracheal route.

**A**) The frequency of CD3+ cells in blood was analysed 24 h after FTY720 administration. Representative dot plots of treated and control animals and the mean frequency of CD3+ T cells in blood are depicted. Differences are significant at p≤0.001 Student’s t test. **B, C**) Mice were immunized with BCG via the s.c. route and revaccinated via the i.t. route 3 weeks later. Mice were challenged 3 weeks after with 200 *M. tuberculosis*  via aerosol. Four weeks post-challenge, mice were sacrificed and CFU were enumerated in lungs (**B**) and spleens (**C**). Individual log_10_ transformed CFU counts are shown with bars indicating mean ±  standard error of the mean (SEM). Differences between groups are significant at * p≤0.05, ** p≤ 0.001 and *** p≤0.001 (one-way ANOVA).
